# RV fibrosis in pulmonary hypertension impact on prognosis; does formal quantitation augment prognosis?

**DOI:** 10.1186/1532-429X-15-S1-P263

**Published:** 2013-01-30

**Authors:** Siva Kumar R Soma, Vishal Goyal, June Yamrozik, Ronald B Williams, Mark Doyle, Srinivas Murali, Raymond L Benza, Robert W Biederman

**Affiliations:** 1 Cardiac MRI, Allegheny General Hospital, Pittsburgh, PA, USA

## Background

Right ventricular (RV) function predicts prognosis in pulmonary hypertension (PH) patients (pts) and right ventricular (RV) failure. Prior studies evaluating of 3D RV ejection fraction (EF) have yielded inconsistent prognostic information. Here we explore the prognostic value of contrast enhanced Cardiac MRI (CMR) in PH (WHO group 1-5) pts.We hypothesize that CMR-Late Gadolinium Enhancement (LGE), a marker for myocardial fibrosis when present in RV or RV insertion points (RVIP), is a predictor of adverse prognosis in PH pts and remains more so when a quantitative analysis is incorporated.

## Methods

A retrospective chart review of PH pts (n=42) who underwent clinically indicated CMR were analyzed. Demographic data: mean age 61 yrs; 26% male; WHO group 1 (55%) group 2 (21%), group 3 (5%) group 4 (14%) group 5 (5%). RV volumetric data were indexed to BSA, and along with RV LGE information (Binary and Manual Quantitation: 5 SD threshold analysis) were correlated with MACE including hospitalization, death, referral/need for lung transplantation and addition/increase in inotropic therapy.

## Results

LGE was positive (+) in 18 pts (43%) and negative (-) in the remaining 24 pts (57%). The predominant MACE events occur in the LGE+ group (78%). Specifically, in LGE+ group, 7 pts (39%) had MACE while 11 pts (61%) did not have MACE. In comparison, the LGE- group had only 2 pts (8%) who had MACE and 22 pts (92%)who did not have MACE, <0.03 for all). The results were similar when WHO group 1 were sub analyzed. In WHO 1 subgroup, 11 pts (48%) were LGE+ and 12 pts (52%) were LGE-. In the LGE+ group 4 pts (36%) had MACE while 7 pts (64% did not. In the LGE- there were no MACE, (<0.04). Fisher's exact test was used for group comparisons. Univariate analysis revealed only RVESVI, RVEF, RVEDVI and MRI LGE predicts MACE. However, via multivariable logistic regression analysis only RVESVI (OR: 1.1, 95%; CI 1.0-1.2) and MRI LGE (OR: 7.0,95%;CI 1.2-39.5) predict MACE.(χ2=22.5,df=2, n=42, p<0.001). In a subset of those +LGE pts who had none-zero fibrosis after threshold quantitiation, no additive value was demonstrable.

## Conclusions

RV-LGE is >seven-fold stronger predictor of MACE than standard CMR metrics in PHTN pts. LGE's role as an independent adverse prognosticator may define the pathophysiologic hallmark in PH pts directly reflecting underlying RV failure due to progressive myocardial fibrosis. However, in limited fashion, quantitation appeared not to add clincal value over visual assessment.

## Funding

Internal

